# New-Onset Mania and Psychosis in Adolescents in the Context of COVID-19 Infection

**DOI:** 10.7759/cureus.24322

**Published:** 2022-04-20

**Authors:** Ryan Meeder, Samicchya Adhikari, Kiela Sierra-Cintron, Kapil Aedma

**Affiliations:** 1 Psychiatry, UnityPoint Health - Methodist Hospital/University of Illinois College of Medicine, Peoria, USA

**Keywords:** covid-19-induced psychosis, new episode psychosis, mania, psychosis, children, covid-19

## Abstract

There is a growing body of evidence that coronavirus disease 2019 (COVID-19) is linked with neuropsychiatric complications such as psychosis and delirium in adults. Much less is known about the neuropsychiatric manifestations of this virus in the child and adolescent population. This case series discusses two unique adolescent patients who presented with new-onset mania and psychosis in the context of an otherwise asymptomatic COVID-19 infection, which raises several questions about whether and how the virus precipitates mania and psychosis, whether these symptoms are transient or predisposes patients to a chronic psychiatric disorder, and confounding variables that may have contributed to the symptoms. These questions can then be points for future research and longitudinal follow-up that can better improve our knowledge about the relationship between this complicated virus and how it affects children psychiatrically.

## Introduction

Coronavirus disease 2019 (COVID-19) has been well known to cause significant pulmonary and respiratory symptoms. In addition, there is a growing body of evidence that this virus is linked with neuropsychiatric complications [1]. Many case reports have been published linking COVID-19 infection with psychosis and delirium in adults [2]. The proposed explanations in adults may also be applicable to children and adolescents [3], although there are much fewer published cases of COVID-19-related psychosis in adolescents. Comparisons between adults and children are also challenging, as fewer cases of severe respiratory infection have been reported in children [4].

The few adolescent cases of psychosis that have been reported were associated with high levels of psychosocial stress [5]. It is well known that the COVID-19 pandemic caused significant stress to adults and children worldwide. In children and adolescents, the stress manifests itself as increased anxiety, irritability, insomnia, clingy behavior due to fear of contracting infection, social isolation, and emotional distress from losing loved ones to the virus [3,5]. One case published in 2021 reported an episode of new-onset mania in a healthy individual after the death of her mother due to COVID-19. This same case series also discussed another adolescent patient with psychosis following academic stress in a virtual setting [5].

COVID-19 is also associated with a wide variety of neuropsychiatric symptoms, such as changes in taste, smell, and focal neuropathies [6]. The proposed explanations of how the virus mediates these neuropsychiatric symptoms include the role of cytokine storms and autoantibody formation during postinfectious periods, but these explanations are based on cases following active symptomatic COVID-19 infection [6]. Another case report of a child patient discusses psychotic symptoms in the context of COVID-19 infection, but this patient also tested positive for anti-NMDA receptor antibody [7]. The cases we present are notable in that despite testing positive for SARS-CoV-2, there was no evidence of symptomatic infection.

Given the paucity of information available regarding the link between neuropsychiatric conditions and COVID-19 in adolescents, this case series may help to add knowledge to the growing literature and guide management and future direction for additional research.

## Case presentation

Case 1

This is a case of a 16-year-old male with a history of depression, anxiety, attention deficit hyperactivity disorder (ADHD), and asthma who presented to the hospital for new-onset mania and psychosis in the context of an otherwise asymptomatic COVID-19 infection. His mother brought him in due to statements that his family was plotting against him and he was being monitored by cameras at home. His family noted irritability, mood lability, insomnia, and auditory hallucinations that his family was conspiring against him. Per family, this started two weeks preceding admission. He tested positive for SARS-CoV-2 via PCR, which was found incidentally due to laboratory tests required for admission per hospital policy. His urine drug screen was negative, but he reported a prior one-year history of marijuana use, which he had stopped two months before this admission. Other basic laboratory tests and imaging were unremarkable, and he did not have any typical COVID-19 symptoms. The patient lived with his biological mother and stepfather. His mother has a history of bipolar disorder and post-traumatic stress disorder. His biological father had a history of substance use disorder. He had also started to struggle academically in school when his symptoms started.

The patient presented with a flight of ideas, disorganized thoughts, mood lability, irritability, and rapid speech on arrival. He exhibited paranoia and delusions such as believing that others were stealing his emotions from him, his mother was stealing his energy, and his mother and stepfather were trying to hurt him. The patient expressed paranoia that his uncle laced his marijuana when he had last used it two months ago. His symptoms, however, started over a month after the cessation of drug use. He was seen responding to internal stimuli and demonstrated intermittent thought blocking during the initial assessment. Per his mother, the patient had only been getting 2-3 hours of sleep for several days despite running and exercising excessively to wear himself down. He had poor oral intake over the last few weeks preceding admission. He had not showered for several days. Although he carried a prior history of depression, anxiety, and ADHD, he had not been on any psychotropic medications for at least three years, and prior medication trials were unknown. This was the patient’s first psychiatric admission, and there was no history of suicide attempts or self-harm.

The patient was started on olanzapine 5 mg twice daily to target psychosis and mania. During his initial hospital course, the patient demonstrated severe paranoia, irritability, thought blocking, and disorganized thoughts. Over the course of hospitalization, olanzapine was titrated to 5 mg daily and 15 mg nightly due to persisting psychosis. He was also started on lorazepam 1 mg nightly to help with severe insomnia, which was tapered and discontinued within one week of discharge. The patient gradually showed improvement with psychosis, irritability, lability, and insomnia with medications and therapy in the inpatient unit. He was no longer seen responding to internal stimuli, and his paranoia improved. He was able to engage more appropriately in assessment, and the family felt he had improved significantly. At the time of discharge, the differential diagnosis included bipolar I disorder, current episode manic with psychotic features, rule out substance-induced mood disorder, and psychotic disorder due to another medical condition (COVID-19 infection). This differential was formulated by a board-certified child and adolescent psychiatrist and psychiatry resident physician working together.

Case 2

This is a case of a 17-year-old male with no prior psychiatric history, no previous inpatient psychiatric admissions, and no prior psychotropic medication trials who presented to the emergency department due to altered mental status. Per reports from family, the patient received Individualized Education Program (IEP) resources at school for developmental delays, although formal psychological testing results, specifically cognitive testing, were not available for review. There was no family history of psychiatric illness. At the time of admission, the patient exhibited bizarre behaviors, disorganized speech and thought content, religious preoccupation, paranoia, and delusions in the context of asymptomatic COVID-19 infection. Initially, the patient’s mother indicated that he was pacing and restless at home, often walking in and out of the house for several hours at a time, unable to stay in one location. He also professed himself as a prophet with the ability to read people’s minds. Several members of the family had also tested positive for COVID-19 infection and were recovering with supportive care. Aside from testing positive for SARS-CoV-2 via PCR, medical workup in the emergency department was largely unremarkable, including basic laboratory tests and head CT. He was admitted to the pediatric floor for further stability and assessment, and child and adolescent psychiatry was consulted.

Initial psychiatric assessment was notable for grandiosity, religious preoccupation, paranoia, and disorganized thoughts. He was impulsive and made comments about possessing an all-seeing eye. The family was present at the bedside and indicated that his presentation was a significant shift from baseline behavior and that he had never exhibited similar behaviors in the past. The family frequently needed to redirect him throughout the assessment, and he often made comments about joining his deceased brother. Throughout his admission, he did require as-needed medications for agitation and disorganized behavior, including olanzapine, haloperidol, lorazepam, and diphenhydramine. Neurology was also consulted who completed additional medical workup, including brain MRI with and without contrast, EEG, and lumbar puncture. The results were unremarkable. This additional medical workup included multiple sclerosis profile, VDRL, CSF West Nile IgG/IgM, Lyme disease, CSF film array PCR panel, CSF angiotensin-converting enzyme, CSF myelin basic protein, CSF Gram stain and culture, CSF protein count, chloride, cell count and glucose, CSF fungal culture, ANA immunoassay screen, and procalcitonin. These results were within normal limits.

He was started on olanzapine and lithium targeting psychosis and mood lability. Target symptoms included paranoia, grandiosity, religious preoccupation, and disorganization. He did require one-to-one sitters throughout admission on both the pediatric and inpatient psychiatry units. He remained on the pediatric medical floor for several days prior to his transfer to inpatient psychiatry. After transfer to inpatient psychiatry, he was discharged approximately 10 days later. He did show improvement regarding impulsivity and was able to tolerate longer interviews without leaving his room abruptly. His parents noticed an improvement in his behaviors. He was resistant to taking psychotropic medications, however, which made his prognosis more guarded. He demonstrated poor insight into his hospital course and treatment. He was ultimately discharged on lithium 300 mg BID for mania and mood stability and olanzapine 7.5 mg BID for psychosis.

Differential diagnoses included bipolar I disorder, current episode manic with psychotic features, psychotic disorder due to another medical condition (COVID-19 infection), and unspecified psychosis per the treatment team, which consisted of a board-certified child and adolescent psychiatrist and psychiatry resident physician. The onset of his asymptomatic COVID-19 infection correlated with the onset of his abnormal and bizarre behaviors. He did have a history of some possible developmental delay, which may also predispose him to delirium secondary to an infection, but he did not show any signs of active infection throughout his admission. Assessment and collateral from his family also indicated that if there was a developmental delay, it was mild.

## Discussion

The differential for these cases remains broad, and with additional data emerging related to COVID-19 infection in adolescents, it will hopefully become clearer. The possible explanations for COVID-19 infection-related psychosis have been proposed primarily based on data obtained in adults. These hypotheses include hematogenous spread across the blood-brain barrier, as a sequela of systemic inflammation, and across the cribriform plate [8]. There is also an association between the risk of developing psychotic disorders with severe infection [9]. Few studies linking infection and psychosis in children and adolescents have been published [10]. This case series may demonstrate a link between asymptomatic COVID-19 infections and psychosis/mania in children and adolescents. The cases presented here, however, are different in that they did not show evidence of severe infection or CNS involvement. Extensive medical workup also supported a primary psychiatric condition, potentially exacerbated or triggered by COVID-19 infection. To our knowledge, cases of new-onset psychosis or mania in children and adolescents in the setting of COVID-19 infection have not been reported. One study from May 2021 did report two cases of pediatric acute-onset neuropsychiatric syndrome that started two weeks after testing positive for COVID-19 infection via PCR; however, these cases reported symptoms consistent with obsessive-compulsive disorder (fear of contamination), irritability, and facial and motor tics rather than the psychosis and mania seen here [11].

As case reports have indicated with adults, there are also some potential confounding factors in the cases discussed. In the first case, the patient did have a history of substance use but was abstinent from cannabinoids for two months before his presentation. Cannabis-associated psychosis has been reported but is typically associated with excessive, frequent, or continuous use. The patient also had a family history of bipolar disorder, which also raises the question of whether his first manic episode was inevitable but simply happened to coincide with his infection in a particularly stressful time amidst the COVID-19 pandemic. Historically, rates of psychosis have increased following pandemics, but there is limited data examining COVID-19 infections in this capacity [9]. Also, the COVID-19 pandemic has placed a significant burden on society from a cultural, social, and psychological perspective. This raises concern that stress-related reactions to the infection are an additional confounding variable. The COVID-19 pandemic places children and adolescents under significant social and family stress [5]. In the second case, there was a complex family dynamic, and the patient’s parents held opposing views regarding the etiology of his symptoms. It seems unlikely, however, to fully explain the symptoms observed in the second case. From the treatment team’s perspective, he showed clear signs of mania and psychosis. Psychosocial stressors including family dynamics and stressors related to the pandemic certainly could have contributed to his first psychotic break, but given his lack of family history and lack of any prior psychiatric or medical history, it certainly raises the question of whether and how much COVID-19 may have also played a role.

While cases of new-onset psychosis in adolescents are difficult to find, many cases have been reported in adults, which may also shed some light on the cases presented here. One case report identified a 43-year-old male patient in Spain with no history of mental illness who presented in a manic episode with psychotic features. He had also received treatment with high-dose methylprednisolone for COVID-19 infection. Additionally, he had a remote history of intermittent cocaine use, both significant confounding variables [12]. While the first case also presented with a possible substance use component (THC), it seems less likely to be a confounding factor given its remote use and cessation two months prior to his presentation. It is also difficult to make direct comparisons to the adolescent cases discussed above as they did not require steroid treatment. High-dose steroid treatment is a well-established risk factor for mania [13].

Several other reported cases in adults also mention antibiotic use as a precipitating factor. A case report looking at adult African patients, who were COVID-19-positive and treated with chloroquine, demonstrated psychotic symptoms in one report and severe anxiety in another. Psychiatric side effects from hydroxychloroquine and chloroquine in the context of COVID-19 infection are also poorly understood. Antibiotic treatment was discontinued, and resolution of hallucinations was reported in 48 hours, and the patient remained asymptomatic after follow-up around two months later [14]. It is possible that antibiotic use, particularly quinolones, increased the risk of the emergence of psychotic symptoms in the setting of COVID-19 infection. This link remains unclear. Furthermore, given the absence of antibiotic treatment in the cases presented, further research is warranted regarding comorbid risk factors for psychosis in the context of COVID-19 infection with antibiotic use.

Another case report discussed a 30-year-old male with no psychiatric history diagnosed with brief psychotic disorder following COVID-19 infection in the context of worsening stress due to quarantine [15]. This study attributed an increased risk for psychotic symptoms due to this patient’s increased anxiety regarding having COVID-19. COVID-19-related concerns were not seen in all cases reported in adults as an additional risk factor for new-onset psychosis. Three patients with new-onset psychosis from New York [16] and one from North Carolina [17] reported very little anxiety surrounding the infection itself. Many cases report patients with a history of depression or anxiety, and the COVID-19 pandemic is a significant environmental stressor. In the two presented cases, one patient did carry a history of ADHD, depression, and anxiety but was incidentally found to have COVID-19. Although the pandemic was not an overt source of stress for the cases in question, the pandemic has no doubt caused extreme anxiety in adults as well as children, which can certainly contribute to a first psychotic break.

Neurological involvement in adult cases has also been viewed as potential evidence for CNS involvement. Some studies have indicated that anosmia suggests CNS involvement in COVID-19 infections [18]. In the cases discussed here, there was no CNS involvement or anosmia. The cases presented had no objective findings of inflammation at the time of admission, despite presenting with significant psychotic symptoms and testing positive for COVID-19. Both patients were assessed by pediatricians who documented normal physical examinations and no concerns for active infection. One study looking at the 2003 SARS epidemic in Hong Kong demonstrated that children were at risk for SARS-CoV but did not identify any significant psychological or psychiatric disturbances [19]. Additionally, SARS-CoV has been isolated from brain tissue in the past, and it would be reasonable to hypothesize that COVID-19 could also present with similar findings, as SARS-CoV and COVID-19 are nearly identical taxonomically [8]. Given the limited data on neural tissue regarding COVID-19 infection, this remains a topic that warrants future investigation.

## Conclusions

COVID-19 remains a significant stressor in both adult and adolescent populations. New-onset psychosis in children and adolescents in the setting of COVID-19 infections is poorly understood. These cases demonstrate some of the first documented cases of an adolescent patient with new-onset psychosis or mania in the setting of an otherwise asymptomatic COVID-19 infection. Several confounding factors that have been present in adult cases, such as steroid use, antibiotic use, psychosocial stressors, isolation, financial hardship, polysubstance use, and recent prior psychotropic medication use, are not significant factors in these patients. This case series raises several important questions. Is there an association between COVID-19 infections and psychosis or mania? Are the mania and psychosis transient in the context of recent infection or do both actually precipitate a chronic psychiatric disorder such as bipolar disorder or schizophrenia? Is COVID-19 infection an important risk factor for childhood schizophrenia or bipolar disorder? The increased frequency of psychosis we have seen in adults suggests that there is a link between the infection and precipitating psychosis. These cases suggest that children and adolescents may also carry this risk, albeit at a lower rate. Longitudinal follow-up of cases like the ones presented above will help further improve our knowledge of the relationship between the virus and psychiatric disorders. As more cases of COVID-19 are discovered, we may see an increased frequency of child or adolescent-onset psychosis or mania. Additionally, with the advent of the COVID-19 vaccination and its focus on the elderly population, the number of children and adolescents who present with new-onset psychosis or mania may increase.
